# Skewed pretransplant lymphocytes subpopulations correlate with opportunistic infection onset within the first two years following kidney transplantation

**DOI:** 10.3389/fimmu.2025.1684313

**Published:** 2025-11-06

**Authors:** Asma Beldi-Ferchiou, Florence Runyo, Florence Canoui-Poitrine, Bastien Peiffer, Benyamin Mattei Dediu, Cécile Maud Champy, Julie Oniusciuk, Giovanna Melica, Cédric Usureau, Nizar Joher, José Cohen, Philippe Grimbert, Caroline Pilon, Marie Matignon

**Affiliations:** 1 Immunology Department, Henri-Mondor/Albert-Chenevier University Hospital, APHP, Créteil, France; 2 Institut National de la Santé et de la Recherche Médicale (INSERM) U955, Institut Mondor de Recherche Biomédicale (IMRB), Université Paris-Est Créteil, Créteil, France; 3 Nephrology and Renal Transplantation Department, Henri-Mondor/Albert-Chenevier University Hospital, Fédération Hospitalo-Universitaire TRUE (InnovaTive theRapy for immUne disordErs), APHP, Créteil, France; 4 Public Health Department, Hôpitaux Universitaires Henri Mondor-Albert Chenevier, Assistance Publique-Hôpitaux de Paris (AP-HP), Créteil, France; 5 Assistance Publique-Hôpitaux de Paris AP-HP, Hôpital Henri Mondor, DMU Medecine, Creteil, France; 6 Urology Department, Henri-Mondor/Albert-Chenevier University Hospital, APHP, Créteil, France; 7 Infectious disease Department, Henri-Mondor/Albert-Chenevier University Hospital, APHP, Créteil, France; 8 Equipe 18 Virus, Hépatologie, Cancer, Inserm U955, Université Paris Est Créteil (UPEC), Créteil, France; 9 Laboratoire d’Immunologie et Histocompatibilité, Hôpital Saint Louis, Paris, France; 10 INSERM UMR976, Institut de Recherche Saint-Louis, Université de Paris-Cité, Paris, France; 11 CIC Biothérapies Department, Henri-Mondor/Albert-Chenevier University Hospital, Fédération Hospitalo-Universitaire TRUE (InnovaTive theRapy for immUne disordErs), APHP, Créteil, France

**Keywords:** kidney transplantation, opportunistic infection, acute rejection, immunomonitoring, NK cells

## Abstract

**Introduction:**

After kidney transplantation (KT), there is no reliable assessment of the immunosuppressive state. We analysed pre-KT T-, B- and NK-cell populations in relation to the occurrence of opportunistic infections (OI) or acute rejection (AR) after KT.

**Methods:**

We included 422 adult KT recipients from 01/2016 to 09/2020. Immune cells were analysed using flow cytometry in 283 recipients before KT in three groups: AR, OI or no event within 24 months after KT.

**Results:**

There were 49 recipients in the OI group, 44 in the AR group and 190 in the control group. Before KT, higher absolute counts and percentages of NK cells (p=0.001 and p=0.007 respectively), elevated absolute counts of plasmablasts and CD21^-^CD38^-^ B cells (age-associated B cells) (p=0.045 and p=0.028 respectively), and a lower proportion of CD3+ T cells (p=0.022) were independently associated with the occurrence of OI within two years following kidney transplantation (KT). In recipients with OI occurring before three months, only absolute count of NK cells before KT remained independently associated with the occurrence of OI (p=0.002). None of the studied immune cell population was associated with AR.

**Conclusion:**

Our results suggest that higher levels of pretransplant NK cells and age-associated B cells are correlated with the occurrence of OI within two years after KT. This result may improve stratification of individualized infectious risk prior to KT.

## Introduction

Kidney transplantation (KT) remains the optimal treatment for end-stage renal disease (ESRD), although certain considerations apply to the elderly population (> 65 years) and those receiving HLA incompatible kidney transplants (1, 2). The primary cause of kidney allograft failure is patient mortality with a functioning allograft (3). The most common causes of patient death include cardiovascular events, infections and malignancy (4). With regard to kidney allografts, the second most common cause of chronic dysfunction and kidney allograft loss is antibody-mediated allograft injury and acute rejection (AR) (5). There is a correlation between severe infections, especially opportunistic infections occurring within one year of transplantation and immunosuppressive therapy (6, 7).

The presence of OI and AR is an indication that immunosuppressive therapy is inappropriate and evidence of over- and under-immunosuppression, respectively. The choice of immunosuppressive therapy is contingent upon the age of the recipient, their history of infections, the presence and type of anti-HLA antibodies, and the level of these antibodies (8). In order to prevent OI, prophylactic strategies have been proposed to be implemented within the first six months following transplantation (9). Lymphopenia at the time of transplantation and during the follow-up period represents a risk factor for OI, necessitating the maintenance of prophylaxis in such patients (7). Furthermore, the net state of immune deficiency following KT can be evaluated by taking into account the immunosuppressive regimen, as well as individual recipient predisposing factors such as diabetes, kidney allograft dysfunction, surgery, or nutritional deficits (9). Concurrently, the principal risk factors for AR following KT are anti-HLA antibodies and donor-specific antibodies (DSA), whether preformed or *de novo* after transplantation (10).

ImmuKnow^®^ is the only FDA-approved clinical test for measuring overall T cell function in transplant recipients thus providing an additional tool to help identify patients at risk for infection and AR. Nevertheless, the manner in which it provides a prediction remains unclear, and the optimal use of the test in KT recipients has yet to be determined (11, 12). An immune risk profile (IRP) based on the immunophenotype of T and NK cells along with CMV serostatus, has been proposed as a surrogate marker of immune senescence, with predictive value for both OI and severe bacterial infection (13). The incorporation of NK cells into the IRP may enhance the accuracy of the prediction (14). Regarding the humoral immune response, pre-transplant serum level of B-cell activating factor (BAFF) has been identified as a risk factor for both AR and *de novo* donor-specific antibodies (DSAs) following transplantation (15). Conversely, a low level of gammaglobulins has been associated with an increased risk of severe infections (16).

Given the limitations of the available data, which preclude a reliable evaluation of the immunosuppressive state and the infection-induced mortality rate after KT, we conducted a prospective, monocentric study with the objective of analysing the capacity of lymphocyte count (B, T and NK cells) and B cell sub-population analysis to predict the first episode of OI or AR within two years after KT.

## Materials and methods

A prospective study analysing pre-transplant lymphocyte population was conducted including all KT recipients who underwent engraftment between April 2016 and September 2020 at Henri Mondor Hospital, AP-HP, Créteil, France. All patients were followed over time to assess post-transplant outcomes. This hypothesis-driven, proof-of-concept study aimed to explore whether pre-transplant lymphocyte populations could serve as potential predictors of post-transplant outcomes.

The exclusion criteria were as follows: a delay between KT and the capacity to analyse cells longer than 24 hours; combined solid organ transplantation or former KT; former exposure to anti-CD20 treatment; inclusion in a drug-testing protocol; and kidney allograft loss before seven days after KT. Prior to KT, all recipients underwent a thorough clinical and biological evaluation to exclude evidence of active infection. An elevated C-reactive protein (CRP) level above 5 mg/L was an exclusion criteria to KT.

The local ethics committee (IRB #00003835) granted approval for the description of the cohort of transplant recipients and the analysis of biomarkers in KT recipients.

An expanded criteria donor (ECD) was defined as a donor over 60 years of age or between 50 and 60 years of age with two of the following three criteria: (i) hypertension; (ii) serum creatinine concentration exceeding 1.50 mg/dL prior to retrieval; and (iii) cerebrovascular cause of brain death. The glomerular filtration rate was estimated (eGFR) utilising the Chronic Kidney Disease Epidemiology Collaboration (CKD-EPI) formula. Delayed graft function (DGF) was defined by dialysis requirement within seven days post-transplant. AR episodes were classified in accordance with the updated Banff classification, which differentiates between acute antibody-mediated rejection (ABMR) and T-cell-mediated rejection (TCMR) (17). The definition of OI is based on existing criteria and is described in detail in the supporting information (**Supplementary Data**) (7). Allograft loss was considered as an eGFR below 10 mL/min/1.73 m² or the commencement of dialysis. All recipients were followed for two years after transplantation, unless death or allograft loss occurred earlier.

The initial event defined the patient group assignment. The primary outcome was AR or OI. The control group was free of AR and OI.

### Endpoints

The primary endpoint was the correlation between the pretransplant immune cells immunophenotype and initial episodes of OI or AR within the first two years post-KT.

Secondary endpoints were as follows: (i) kidney allograft survival, (ii) patient survival, (iii) eGFR, (iv) occurrence of AR, (v) occurrence of *de novo* DSA (dnDSA), (vi) occurrence of OI and (vii) correlation between episodes of OI or AR from three months to two years after KT and the evolution of immune cells between the day of transplant and three months after KT.

### Flow cytometry analysis of B, T and NK cells sub populations

Blood samples were prospectively collected immediately before and three months after KT. Samples were collected in ethylenediamine tetra acetic acid (EDTA) tubes at room temperature. They were analysed within two hours in the immune-biology department of Henri-Mondor Hospital (France). B-cell subsets were analyzed using standardized DuraClone IM B Cells technology (Beckman-Coulter) containing a pre-formulated dry reagent comprising eight monoclonal antibodies (IgD-FITC, CD21-PE, CD19-ECD, CD27-PC7, CD24-APC, CD38-APCA750, IgM-Pacific Blue and CD45-Krome Orange). The whole blood (300 µL) was washed twice in 1X phosphate-buffered saline (PBS), the supernatant was discarded and the pellet resuspended in an additional 300 µL of PBS. We added 100 µL of washed blood to the DuraClone IM B reagent tube and incubated for 15 minutes at RT in the dark. After lysing red blood cell with VersaLyse Solution (Beckman-Coulter), samples were acquired on a Navios cytometer (Beckman-Coulter) and analysed with Kaluza (Beckman-Coulter). An independent TCD3+, TCD4+, TCD8+, B (CD3-CD19+) and NK cells (CD3- CD56+ CD16+) absolute count was performed on whole blood using the fully automated AQUIOS cytometer ((Beckman-Coulter) and the ready to use AQUIOS Tetra-1 panel (CD45-FITC/CD4-RD1/CD8-ECD/CD3-PC5) and AQUIOS Tetra-2 panel (CD45-FITC/(CD56+CD16)-RD1/CD19-ECD/CD3-PC5) both from (Beckman-Coulter). The results were expressed as cell numbers per microliter and as a percentage. Gating strategy is illustrated in **Supplementary Figure S1**. All the details of the antibodies clones used for B cells subpopulation study and AQUIOS lymphocytes count are available in **Supplementary Table S1**.

### Anti-HLA antibody screening

DNA typing was conducted on both donors and recipients at the HLA-A, HLA-B, Cw, HLA-DR, and HLA-DQ loci. Serum samples were taken from the donor and recipient prior to and following the KT. Samples were analysed for preformed DSA and dnDSA targeting donor HLA antigens, using high-resolution Luminex SAB assay technology (One Lambda, Inc., Canoga Park, CA) on a Luminex platform. Beads with a mean fluorescence intensity (MFI) above 500 were considered positive.

### Statistical analysis

The data were described using the relevant statistical conventions. A univariable analysis compared patients who did not experience an event with those who experienced AR or OI within 24 months following KT. The no-event group was the reference point. The most appropriate statistical test was used for quantitative variables: one-way analysis of variance (ANOVA), Student’s t-test, or Kruskal-Wallis test. Categorical variables were tested with a Chi-squared or Fisher’s exact test. P-values were corrected by the Benjamini-Hochberg procedure when needed. A correlation matrix was constructed to evaluate collinearity between quantitative variables. Univariable and multivariable logistic regression were conducted based on significant variables and variables with a p-value of less than 0.10. The multivariable analysis involved aligning biological data to clinical data. In the second stage, only uncorrelated biological data was taken in account. To compare quantitative variables over time, the paired t-test was used.

No data were imputed for missing values. Tests were bilateral, and p-value < 0.05 indicated significance. Analyses were performed using R (version 4.2.0).

## Results

**Figure 1:**
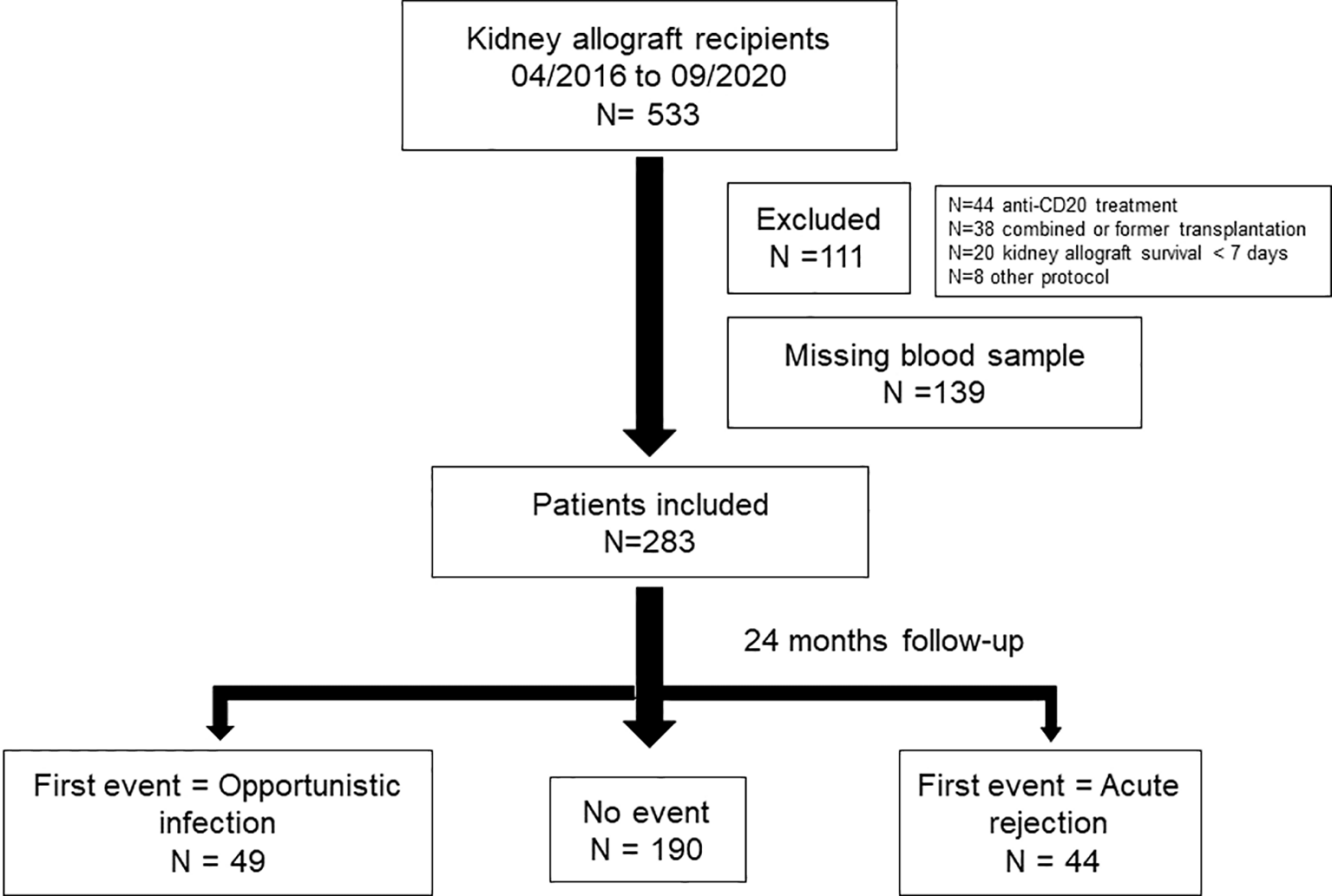
Flow chart of the patients included in the study N=533 patients were kidney engrafted between 04/2016 and 09/2020. Among those, N = 111 patients were excluded, N = 44 because of exposure to anti-CD20 treatment before transplantation, N = 38 because of combined transplantation, N = 20 because of kidney allograft survival below than 7 days and N = 8 because of another immunosuppressive protocol. Among the N = 422 included, N = 139 have missing samples. A total of N = 283 patients were included in the study and followed 24 months. N = 49 kidney allograft recipients were included in the OI group, N = 44 in the AR group. Control group included N = 190 kidney allograft recipients.

**Figure 1** shows a flowchart of the patients included. Adult kidney allograft recipients were enrolled at the time of KT (N = 422). The unavailability of blood samples in 139 recipients limited the analysis to 283 patients. The two groups were similar in terms of characteristics examined (**Supplementary Table S2**). The control group comprised 190 KT recipients without AR or OI.

Within the first two years after transplantation, 49 patients experienced an OI episode 6.9 [1.8;12.9] months after KT, while 44 had an AR episode 3.0 [0.6;8.9] months after KT. Among the 44 cases of AR, 24 (54.5%) were TCMR, 12 (27.3%) ABMR, and 8 (18.2%) mixed. Among the OI episodes, 26 (53.1%) were viral, 18 (36.7%) were bacterial, and 5 (10.2%) were fungal.

**Table 1** illustrates recipients and KT characteristics. Compared to the control group, recipients from OI group were significantly older (p=0.047). No clinical factors were identified as being associated with the initial episode of AR within 24 months following KT. At the completion of the follow-up period, the control group exhibited significantly superior kidney allograft function compared to the AR and OI groups (p < 0.001 and p = 0.001, respectively). Nevertheless, the mortality rates and allograft survival were comparable between the three groups.

**Table 1:** Recipients characteristics and kidney transplantation evolution.

Variables	All cohort	Control	First acute rejection within 24 months after kidney transplantation	First opportunistic infection within 24 months after kidney transplantation	p-value control vs. AR	p-value control vs.OI
Patients, N (%)	283 (100)	190 (67.1)	44 (15.5)	49 (17.3)		
Recipients characteristics						
Age, years, median [IQR]	55.3 [44.9;65.7]	53.3 [43.0;64.6]	56.2 [44.6;67.2]	58.7 [52.0;67.8]	0.428	0.047
Sex, Female, N (%)	103 (36.4)	64 (33.7)	17 (38.6)	22 (44.9)	0.689	0.590
Diabetes before KT, N (%)	72 (25.4)	46 (24.2)	11 (25.0)	15 (30.6)	1.000	1.000
Dialysis, N (%)	254 (90.7)	165 (88.2)	41 (93.2)	48 (98.0)	0.429	0.163
Hemodialysis, N (%)	232 (91.3)	149 (90.3)	36 (87.8)	47 (97.9)	0.576	0.192
Dialysis duration (months), median [IQR]	38.9 [22.7;62.5]	39.2 [21.9;62.0]	36.1 [23.1;48.4]	42.8 [28.2;70.3]	0.443	0.320
Donor characteristics						
Living donor, N (%)	38 (13.4)	30 (15.8)	3 (6.82)	5 (10.2)	0.580	0.671
Extended Criteria Donor, N (%)	142 (50.2)	89 (46.8)	23 (52.3)	30 (61.2)	0.630	0.306
Age, years, median [IQR]	57.0 [47.5;66.5]	56.5 [46.0;65.0]	57.0 [50.0;73.2]	62.0 [51.0;68.0]	0.279	0.128
eGFR, ml/min/1.73m², median [IQR]	91.6 [67.2;106]	92.8 [67.7;106]	88.1 [56.6;111]	91.3 [71.3;104]	0.926	0.926
Kidney transplant characteristics						
Donor specific anti-HLA antibodies, N (%)	65 (23.8)	41 (22.3)	10 (23.8)	14 (29.8)	0.993	0.993
Delayed graft function, N (%)	82 (29.0)	48 (25.3)	15 (34.1)	19 (38.8)	0.475	0.268
Immunosuppressive therapy						
Induction, N (%)	282 (99.6)	189 (99.5)	44 (100)	49 (100)	1.000	1.000
Basiliximab, N (%)	108 (38.2)	77 (40.5)	16 (36.4)	15 (30.6)	0.736	0.736
Antithymocyte globulin, N (%)	174 (61.5)	112 (58.9)	28 (63.6)	34 (69.4)	0.714	0.714
Maintenance						
Calcineurin inhibitors, N (%)	280 (98.9)	188 (98.9)	43 (97.7)	49 (100)	0.710	1.000
Mycophenolate mofetil, N (%)	195 (68.9)	125 (65.8)	31 (70.5)	39 (79.6)	0.679	0.277
Belatacept, N (%)	5 (1.77)	4 (2.11)	1 (2.27)	0 (0.00)	1.000	0.876
mTOR inhibitors, N(%)	89 (31.4)	65 (34.2)	13 (29.5)	11 (22.4)	0.679	0.481
Steroids, N (%)	283 (100)	190 (100)	44 (100)	49 (100)	.	.
Within 12 months after transplantation						
Kidney allograft loss, N (%)	4 (1.41)	2 (1.05)	1 (2.27)	1 (2.04)	0.749	0.749
Patient death, N (%)	13 (4.59)	10 (5.26)	2 (4.55)	1 (2.04)	1.000	0.902
eGFR, ml/min/1.73m², median [IQR]	45.5 [32.1;58.2]	50.0 [35.9;60.4]	38.1 [25.6;49.2]	35.4 [28.2;47.7]	0.001	<0.001
Within 24 months after transplantation						
Kidney allograft loss, N (%)	9 (3.18)	4 (2.11)	3 (6.82)	2 (4.08)	0.375	0.665
Patient death, N (%)	26 (9.19)	18 (9.47)	3 (6.82)	5 (10.2)	0.792	0.792
eGFR, ml/min/1.73m², median [IQR]	45.5 [32.8;58.9]	48.7 [37.2;60.0]	38.6 [22.0;52.0]	36.3 [23.8;54.3]	0.010	0.010

**Figure 2:**
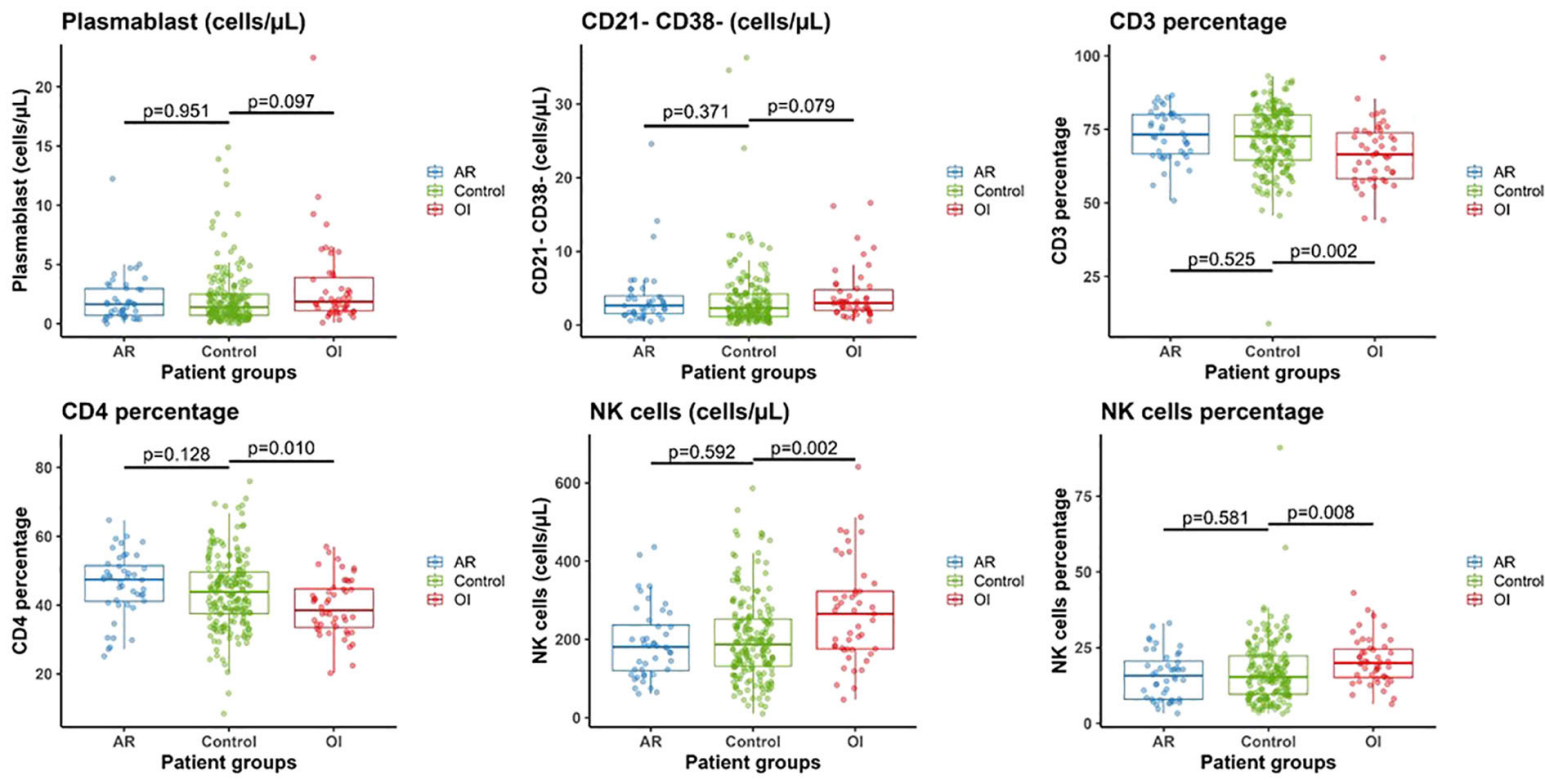
Comparison of B cells subsets, T cells and NK cells in control (n=190), acute rejection (AR) (n=44) and opportunistic infection (OI) (n=49) groups at the time of kidney transplantation.

**Table 2** and **Figure 2** details B, T and NK cells profiles at the time of KT, stratified by group. There was no discernable pre-transplant difference in cellular composition of recipients who later experienced AR. Lower proportions of CD3 and CD4 were observed in patients with OI (p = 0.002 and p = 0.010, respectively), while the CD4/CD8 ratio remained similar. In contrast, patients with OI had significantly higher NK cell counts and proportions (p = 0.002 and p = 0.008, respectively). Regarding different types of OI, NK cells absolute number was similar in viral, bacterial and fungal infections groups (222 [176;405], 232 [171;293] and 304 [294;308] respectively p=0.223) while NK cell percentage was significantly higher in viral infections compared to bacterial and fungal infections groups (21.9 [18.1;27.8], 15.7 [14;20.2] and 18.7 [18.2;23.3] respectively p=0.04).

**Table 2:** Immune cells at the time of transplantation.

Variables	Control	First acute rejection within 24 months after kidney transplantation	First opportunistic infection within 24 months after kidney transplantation	p-value three groups	p-value control vs. AR	p-value control vs.OI
Patients, N (%)	190 (67.1)	44 (15.5)	49 (17.3)			
B cells						
Total, CD19+						
Absolute number, median [IQR]	106 [70.0;176]	114 [74.8;202]	114 [76.0;222]	0.482	0.636	0.636
Percentage, median [IQR]	9.65 [6.38;13.7]	9.55 [7.68;13.8]	11.2 [6.52;16.3]	0.504	0.604	0.604
Double negative memory (IgD- CD27-)						
Absolute number, median [IQR]	3.92 [2.24;7.12]	3.84 [2.02;5.68]	3.82 [2.53;7.51]	0.604	0.574	0.574
Percentage, median [IQR]	3.76 [2.20;5.84]	3.29 [2.07;4.98]	3.30 [2.15;5.69]	0.721	0.730	0.730
Unswitched memory (IgD+ CD27+)						
Absolute number, median [IQR]	14.3 [8.57;22.4]	15.5 [8.62;21.9]	12.0 [8.62;20.9]	0.650	0.670	0.687
Percentage, median [IQR]	13.0 [8.56;17.8]	11.2 [8.18;21.9]	10.8 [8.08;14.6]	0.250	0.995	0.289
Switched memory (IgD- CD27+)						
Absolute number, median [IQR]	15.5 [9.54;24.8]	14.4 [8.94;29.3]	18.6 [11.0;27.8]	0.619	0.989	0.751
Percentage, median [IQR]	15.3 [8.85;24.5]	12.7 [9.51;24.9]	15.0 [8.93;20.8]	0.766	0.732	0.732
Naïve (IgD+ CD27-)						
Absolute number, median [IQR]	67.2 [36.2;123]	69.2 [49.9;136]	78.0 [45.2;177]	0.297	0.518	0.421
Percentage, median [IQR]	65.5 [51.2;77.3]	71.7 [49.0;77.9]	71.1 [60.6;79.8]	0.424	0.754	0.549
Transitional (CD24high CD38high)						
Absolute number, median [IQR]	4.60 [2.30;10.8]	3.96 [1.86;9.85]	6.45 [3.43;16.3]	0.118	0.642	0.113
Percentage, median [IQR]	4.59 [2.68;7.08]	4.17 [1.73;7.22]	6.02 [3.71;7.90]	0.187	0.435	0.188
Plasmablasts (CD24- CD38high CD27high)						
Absolute number, median [IQR]	1.39 [0.70;2.50]	1.63 [0.71;2.96]	1.85 [1.09;3.89]	0.096	0.951	0.097
Percentage, median [IQR]	1.29 [0.66;2.51]	1.21 [0.69;1.98]	1.45 [0.91;2.79]	0.361	0.637	0.335
New memory						
Absolute number, median [IQR]	1.85 [0.93;3.41]	2.05 [1.00;3.24]	2.05 [1.07;5.00]	0.583	0.973	0.667
Percentage, median [IQR]	1.65 [0.92;3.39]	1.68 [1.04;2.74]	1.92 [0.97;3.37]	0.942	0.992	0.992
CD21- CD38-						
Absolute number, median [IQR]	2.28 [1.12;4.21]	2.65 [1.53;3.96]	2.97 [2.00;4.75]	0.077	0.371	0.079
Percentage, median [IQR]	1.86 [1.10;4.30]	2.13 [1.32;3.87]	2.52 [1.37;4.11]	0.624	0.645	0.645
T cells						
CD3+						
Absolute number, median [IQR]	846 [646;1124]	904 [641;1232]	770 [655;1079]	0.618	0.639	0.639
Percentage, median [IQR]	72.7 [64.6;80.0]	73.3 [66.6;80.1]	66.6 [58.3;73.8]	0.001	0.525	0.002
CD4+						
Absolute number, median [IQR]	497 [381;711]	581 [420;758]	466 [374;612]	0.165	0.273	0.273
Percentage, median [IQR]	43.9 [37.5;49.7]	47.4 [41.1;51.5]	38.5 [33.6;44.7]	0.003	0.128	0.010
CD8+						
Absolute number, median [IQR]	292 [198;431]	308 [200;469]	312 [196;428]	0.963	0.950	0.950
Percentage, median [IQR]	24.4 [19.8;32.1]	25.6 [17.6;31.0]	26.0 [17.8;30.9]	0.932	0.994	0.994
CD4/CD8	1.65 [1.26;2.46]	1.81 [1.35;2.72]	1.62 [1.15;2.16]	0.384	0.431	0.431
NK cells						
Absolute number, median [IQR]	187 [132;252]	181 [120;237]	265 [176;323]	0.001	0.592	0.002
Percentage, median [IQR]	15.3 [9.62;22.3]	15.7 [8.00;20.6]	19.9 [15.2;24.5]	0.006	0.581	0.008

Only age was independently associated to the occurrence of OI episode (**Table 3a**). Biological variables at KT were incorporated into a multivariable analysis model, adjusted for recipient age (donor age not considered due to correlation), DSA before transplantation, MMF treatment and DGF (**Table 3b**). Increased percentage of CD3+ and CD4+ T cells was independently linked to a lower risk of OI. A higher absolute count of plasmablasts and CD21^-^CD38^-^ B cells, as well as both a higher absolute number and percentage of NK cells, were independently associated with an increased risk of OI. The models were corrected according to the correlation of the biological data (**Table 3c**). The initial model demonstrated that an elevated CD21-CD38- B-cell count and a heightened NK cell percentage were independently associated with an increased risk of OI occurrence. The second model demonstrated that a lower percentage of CD3+ T cells and a higher number of plasmablasts were independently associated with the occurrence of OI.

Table 3Univariable and multivariable analysis of risk factors for opportunistic infection within 24 months after transplantation.

**Table 3.a:** Univariable and multivariable logistic regression including clinical variables.

Variables	Control	OI	Univariable analysis		Multivariable analysis	
			OR [95% CI]	p-value	OR [95% CI]	p-value
Recipient age, years, mean (SD)	53.2 (14.1)	58.5 (12.3)	1.03 (1.01-1.06)	0.018	1.03 (1.00-1.05)	0.032
Anti-HLA DSA before transplantation, N (%)	41 (22.3)	14 (29.8)	1.48 (0.71-2.98)	0.283	1.21 (0.56-2.54)	0.624
Mycophenolate Mofetil	125 (65.8)	39 (79.6)	2.03 (0.98-4.53)	0.067	2.15 (0.97-5.17)	0.070
Delayed graft function, N (%)	48 (25.3)	19 (38.8)	1.87 (0.96-3.61)	0.063	1.58 (0.77-3.16)	0.201

**Table 3b:** Univariable and multivariable logistic regression including biological variables.

Variables	Control	OI	Univariable analysis		Multivariable analysis	
			OR [95% CI]	p-value	OR [95% CI]	p-value
B cells						
Plasmablast, cells/µL (log)**, mean (SD)	0.4 (0.9)	0.8 (0.9)	1.53 (1.07-2.21)	0.023	1.54 (1.04-2.32)	0.035
CD21- CD38-, cells/µL, (log)**, mean (SD)	0.8 (1.0)	1.1 (0.7)	1.49 (1.05-2.15)	0.030	1.55 (1.06-2.31)	0.025
T cells						
CD3+, (%)	71.8 (10.9)	66.8 (10.4)	0.96 (0.93-0.99)	0.007	0.97 (0.93-1.00)	0.026
CD4+ (%)	43.9 (10.7)	39.5 (8.3)	0.96 (0.93-0.99)	0.010	0.96 (0.92-0.99)	0.016
NK cells						
percentage, (%) (log)**	2.7 (0.6)	3.0 (0.4)	2.65 (1.41-5.28)	0.004	2.52 (1.25-5.32)	0.012
absolute number, (cells/µL) (sqrt)***	13.7 (3.8)	15.9 (4.0)	1.21 (1.07-1.27)	0.001	1.17 (1.06-1.29)	0.001

The analysis was adjusted for recipient age, DSA before transplantation, MMF treatment and DGF

**log: log transformation applied to the numerical variable

***sqrt: square root transformation applied to the numerical variable

**Table 3c:** Univariable and multivariable logistic regression including biological variables corrected for inter-variable correlations.

Variables	Control	OI	Univariable analysis		Multivariable analysis	
			OR [95% CI]	p-value	OR [95% CI]	p-value
Model 1						
B cells						
CD21- CD38-, cells/µL, (log)**, mean (SD)	0.8 (1.0)	1.1 (0.7)	1.49 (1.05-2.15)	0.030	1.59 (1.06-2.43)	0.028
NK cells						
percentage, (%) (log)**	2.7 (0.6)	3.0 (0.4)	2.65 (1.41-5.28)	0.004	2.95 (1.39-6.70)	0.007
Model 2						
B cells						
Plasmablast, cells/µL (log)**, mean (SD)	0.4 (0.9)	0.8 (0.9)	1.53 (1.07-2.21)	0.023	1.51 (1.02-2.29)	0.045
T cells						
CD3+, (%)	71.8 (10.9)	66.8 (10.4)	0.96 (0.93-0.99)	0.007	0.96 (0.93-0.99)	0.022

**log: log transformation applied to the numerical variable

***sqrt: square root transformation applied to the numerical variable

In order to assess whether the presence of DSAs at KT was associated with the occurrence of OI and/or AR, a subsequent analysis was performed. DSAs were detected in 41 recipients (22.3%) in the control group, 10 (23.8%) in the AR group, and 14 (29.8%) in the OI group. No interactions were identified (**Supplementary Table S3**). Importantly, among the 104 DSA-negative recipients at baseline, 11.5% (n = 12) developed dnDSAs within two years post-KT. Donor age was the only variable associated with dnDSA absence, being higher in patients who remained dnDSA-negative (p = 0.048) (**Table 4**).

**Table 4:** Comparison of recipients (clinical and biological data) with and without *de novo* DSA at 24 months.

Variables	No M24 *de novo* DSA	M24 *de novo* DSA	p-value
Recipients, N (%)	92 (88.5)	12 (11.5)	
Clinical data			
Recipients characteristics			
Age, years, median [IQR]	54.2 [42.7;63.5]	47.5 [42.0;50.9]	0.080
Sex, Female, N (%)	35 (38.0)	2 (16.7)	0.205
Diabetes before KT, N (%)	20 (21.7)	2 (16.7)	1.000
Dialysis, N (%)	81 (88.0)	9 (75.0)	0.203
Hemodialysis, N (%)	74 (91.4)	8 (88.9)	0.585
HIV+, N (%)	2 (2.2)	0 (0.0)	1.000
HCV+, N (%)	4 (4.3)	0 (0.0)	1.000
Donor characteristics			
Living donor, N (%)	14 (15.2)	4 (33.3)	0.215
Extended Criteria Donor, N (%)	45 (48.9)	4 (33.3)	0.478
Age, years, mean (SD)	56.5 [46.8;66.0]	50.0 [41.5;57.0]	0.048
eGFR, ml/min/1.73m², median [IQR]	91.6 [70.3;106.1]	97.5 [71.8;107.8]	0.535
Kidney transplant characteristics			
Delayed graft function, N (%)	23 (25.0)	3 (25.0)	1.000
Immunosuppressive therapy			
Induction, N (%)	91 (98.9)	12 (100)	1.000
Basiliximab, N (%)	47 (51.1)	6 (50.0)	1.000
Antithymocyte globulin, N (%)	44 (47.8)	6 (50.0)	1.000
Maintenance			
Calcineurin inhibitors, N (%)	92 (100)	12 (100)	
Mycophenolate mofetil, N (%)	51 (55.4)	6 (50.0)	0.962
Belatacept, N (%)	2 (2.2)	0 (0.0)	1.000
mTOR inhibitors, N(%)	41 (44.6)	6 (50.0)	0.962
Steroids, N (%)	92 (100)	12 (100)	
Biological data			
B cells			
Total, CD19+			
Absolute number, median [IQR]	98.0 [70.0;160]	118 [67.8;344]	0.435
Percentage, median [IQR]	9.20 [6.45;13.1]	11.2 [5.28;18.0]	0.475
Double negative memory (IgD- CD27-)			
Absolute number, median [IQR]	3.50 [1.99;6.65]	3.96 [2.89;4.94]	0.744
Percentage, median [IQR]	3.52 [1.99;5.53]	3.09 [1.83;5.84]	0.625
Unswitched memory (IgD+ CD27+)			
Absolute number, median [IQR]	13.8 [7.97;22.6]	14.3 [8.98;23.8]	0.711
Percentage, median [IQR]	13.6 [9.03;18.2]	11.6 [8.47;18.3]	0.727
Switched memory (IgD- CD27+)			
Absolute number, median [IQR]	15.2 [9.45;24.8]	14.9 [13.1;22.7]	0.975
Percentage, median [IQR]	16.0 [9.64;25.7]	12.7 [6.24;20.1]	0.344
Naïve (IgD+ CD27-)			
Absolute number, median [IQR]	64.3 [37.1;106]	90.7 [44.3;189]	0.319
Percentage, median [IQR]	64.9 [50.8;77.6]	66.3 [53.8;80.1]	0.607
Transitional (CD24high CD38high)			
Absolute number, median [IQR]	4.44 [2.49;8.40]	5.17 [3.24;24.6]	0.339
Percentage, median [IQR]	4.52 [2.77;6.96]	5.76 [4.03;9.28]	0.199
Plasmablasts (CD24- CD38high CD27high)			
Absolute number, median [IQR]	1.32 [0.64;2.34]	1.55 [0.74;3.61]	0.596
Percentage, median [IQR]	1.39 [0.64;2.63]	2.12 [0.42;3.85]	0.742
New memory			
Absolute number, median [IQR]	1.56 [0.79;3.23]	2.00 [0.84;3.20]	0.747
Percentage, median [IQR]	1.46 [0.79;3.40]	1.40 [0.80;2.77]	0.829
CD21- CD38-			
Absolute number, median [IQR]	2.10 [1.11;3.31]	1.90 [0.66;3.96]	0.633
Percentage, median [IQR]	1.79 [1.11;3.75]	1.59 [1.01;2.50]	0.444
T cells			
CD3+			
Absolute number, median [IQR]	846 [664;1180]	889 [706;1164]	0.793
Percentage, median [IQR]	75.1 [68.3;81.3]	67.4 [64.8;77.2]	0.100
CD4+			
Absolute number, median [IQR]	515 [407;734]	566 [442;713]	0.809
Percentage, median [IQR]	46.7 [39.0;52.1]	42.2 [39.2;45.0]	0.206
CD8+			
Absolute number, median [IQR]	291 [190;432]	312 [224;436]	0.821
Percentage, median [IQR]	23.8 [19.1;32.5]	23.8 [19.9;28.6]	0.677
CD4/CD8	1.79 [1.35;2.62]	1.65 [1.50;2.20]	0.821
NK cells			
Absolute number, median [IQR]	184 [132;237]	165 [121;257]	0.781
Percentage, median [IQR]	15.3 [10.8;19.3]	11.8 [8.70;23.0]	0.709

Our cohort was then stratified according to whether the event occurred before or after three months following KT. Before three months (**Supplementary Table S4**), 23 patients exhibited AR, while 17 had OI. The clinical data at KT were comparable. In recipient with OI occurring before three months, only absolute count of NK cells before KT remained independently associated with OI (p=0.002). No significant differences were observed in B cells (**Supplementary Table S4**).

We identified 21 cases of AR and 32 of OI occurring after three months (**Supplementary Table S5**). Baseline clinical data did not differ between groups. The occurrence of OI was significantly associated with reduced CD3^+^ T cells and elevated NK cells proportion before transplantation (p = 0.006 and p = 0.032 respectively). Among these patients, immunological data at M3 showed that OI group exhibited significantly lower proportions and absolute counts of CD3^+^ T cells (p = 0.034 and p = 0.010, respectively), as well as reduced counts of both CD4^+^ and CD8^+^ T cells (p = 0.024 and p = 0.005, respectively) (**Supplementary Table S6**) while NK and B cell populations was similar in all groups.

## Discussion

We investigated here, in a large cohort of KT recipients, the association between pretransplant immune blood cells phenotype and the occurrence of OI or AR within the first two years following KT. Our findings indicate that an increased risk of OI within the first two years after KT was associated with a skewed profile of pretransplant lymphocyte subpopulations, specifically, higher absolute counts and percentages of NK cells, elevated absolute counts of plasmablasts and CD21^-^CD38^-^ B cells, and a lower proportion of CD3+ and CD4+ T cells. In contrast, no lymphocyte subset was significantly associated with the risk of AR.

OI and AR are two clinical situations paradigmatic of the immunosuppressive therapy; OI is probably related to excessive immunosuppression and AR too weak. We stratified KT recipientsbased on the first occurring event (OI or AR), as such events are highly likely to prompt subsequent adjustments to immunosuppressive treatment. We confirmed that donor and recipient older age are at greater risk of OI (7). No other clinical data has been isolated to predict OI or AR confirming the need for alternative biomarkers. In our study, the presence of DSA was not associated with AR, which may be explained by the fact that our analysis was limited to the first two years post-transplantation.

In addition, the estimated glomerular filtration rate (eGFR) was lower in the OI group compared to controls at both 12 and 24 months post-transplant, with a similar trend in the AR group. However, since these measurements were obtained at fixed time points, it remains difficult to determine whether reduced eGFR represents a cause or a consequence of OI. Nevertheless, impaired renal function may contribute to increased susceptibility to infections after transplantation, underscoring the importance of further studies to elucidate this relationship.

Only OI group had a different distribution of the lymphocyte subpopulations, compared to the control group with an independent association between a lower proportion of T cells (CD3+ and CD4+) and a higher proportion of NK cells and OI. A low number of T cells before and following KT has already been described to favor OI (7). Interestingly, a recent report highlighted an association between high NK cells CD56^dim^ frequency before kidney pediatric transplantation and risk of viral infection after transplantation (18). NK cells frequency increases with age, accompanied by a shift in subset distribution, particularly an increased proportion of CD56^dim^ NK cells. These age-related changes may be associated to immune senescence, altered NK cytokine profile, NK decline proliferative capacity and decrease of NK-dependent viral immune surveillance and elimination (19–21). However, our multivariable model was adjusted for recipients age and high NK cells level remained independently associated with OI. Another clinical factor that could have influenced NK cell counts is the use of mycophenolate mofetil (MMF), given its immunosuppressive properties. However, in our study, MMF use did not differ significantly between groups and was not associated with OI in either univariable or multivariable models. In addition, our biological models were adjusted for MMF use to account for its possible confounding effect. To further explore our results, a deeper analysis of NK cells repertoire and functions is warranted to clarify the underlying associations and pathophysiological mechanisms. Notably, polymorphisms in *KLRC2*—the gene encoding the C-type lectin receptor NKG2C have been associated with ABMR activity after KT (22).

To explain this unexpected association between NK cells and OI, we could propose two main hypotheses. Firstly, NK and T cells may compete for common cytokines such as IL-2, IL-15, and IFN-γ. In patients with low T cell counts prior to transplantation, the relative abundance of cytokines may favor NK cell activation, while limiting T cell responses, thereby facilitating the development of OI (23). Secondly, NK cells are known to modulate T cell function during viral infections. In the context of KT, T cells may become highly activated, and NK cells could suppress T cell responses by targeting and eliminating activated T cells, ultimately impairing antiviral immunity and increasing the risk of infection as already reported in murine models. NK cells can kill both activated CD4+ and CD8+ T cells, thereby dampening the immune response and impairing viral clearance (24, 25). In humans, antigen-activated T cells that express stress-induced ligands—such as MICA and the UL16-binding proteins (ULBP1, ULBP2, ULBP3)—can be recognized and lysed by NK cells through engagement of the NKG2D receptor (26).

Regarding B cells, prior to KT, our results did not show significant differences in the total number or percentage of B cells subsets between control group and the AR group. Regarding OI group, higher absolute count of CD21-CD38- B cells and plasmablasts subpopulation were independently associated with the occurrence of OI. The CD21-CD38- B cells is a subpopulation of memory B cells that lacks the expression of complement receptor 2 (CR2) (27). This rare population expands with age and in chronic infection, inflammatory conditions and common variable immunodeficiency. It has been termed age-associated B cells (ABCs) (27). Higher level of ABCs has been correlated with poor vaccination responses and lower levels of memory B cells (28). Further analysis should be performed to better understand the correlation with OI after KT and their evolution after immunosuppressive treatment.

In conclusion, our results suggest that higher levels of NK cells and ABCs before KT are independently correlated with OI occurrence within two years following transplantation. NK cell function and subpopulations before transplantation and after immunosuppressive therapy should be further investigated. Elevated pre-transplant levels of NK cells and age-associated B cells may serve as biomarkers to stratify risk of OI before KT and thereby to guide personalized immunosuppressive strategies. In particular, the preferential use of a combination of calcineurin inhibitors (CNIs) and mTOR inhibitors—previously associated with a reduced incidence of OI after kidney transplantation—could be considered in high-risk individuals (29). One limitation of our study is the lack of detailed pre-transplant data regarding the proportion of recipients with previous immunosuppression. Additionally, information on past infection history was not comprehensively collected, which could have provided further insights into the variability of NK cell counts observed in our cohort. Future research should aim to incorporate more extensive pre-transplant data to better elucidate the underlying factors influencing immune responses and post-transplant outcomes.

Taken together, our findings highlight the need for prospective validation in an independent cohort to confirm their predictive value and potential clinical utility.

## Data Availability

The original contributions presented in the study are included in the article/**Supplementary Material**. Further inquiries can be directed to the corresponding author.

## Supplementary material

Supplementary Figure 1 Blood flow gating strategy

Supplementary Table 1 List of clones and provides of antibodies used for flow cytometry analysis

Supplementary Table 2 Comparison of the two groups with and without available biological sample for immune analysis

Supplementary Table 3 Interactions between variables and DSA at the time of KT

Supplementary Table 4 clinical and biological (immune cells) characteristics at the time of KT of recipients with AR or OI occurring before three months after KT

Supplementary Table 5 clinical and biological (immune cells) characteristics at the time of KT of recipients with AR or OI occurring after three months

Supplementary Table 6 Immune cells characteristics at M3 of KT recipients with AR or OI occurring after three months

## References

[1] Manook M Koeser L Ahmed Z Robb M Johnson R Shaw O . Post-listing survival for highly sensitised patients on the UK kidney transplant waiting list: a matched cohort analysis. Lancet (2017) 389:727−34. doi: 10.1016/S0140-6736(16)31595-1, PMID: 28065559

[2] Hellemans R Kramer A De Meester J Collart F Kuypers D Jadoul M . Does kidney transplantation with a standard or expanded criteria donor improve patient survival? Results from a Belgian cohort. Nephrol Dial Transplant (2021) 36:918−26. doi: 10.1093/ndt/gfab024, PMID: 33650633 PMC8075371

[3] Awan AA Niu J Pan JS Erickson KF Mandayam S Winkelmayer WC . Trends in the causes of death among kidney transplant recipients in the United States (1996-2014). Am J Nephrol (2018) 48:472−81. doi: 10.1159/000495081, PMID: 30472701 PMC6347016

[4] Coemans M Süsal C Döhler B Anglicheau D Giral M Bestard O . Analyses of the short- and long-term graft survival after kidney transplantation in Europe between 1986 and 2015. Kidney Int (2018) 94:964−73. doi: 10.1016/j.kint.2018.05.018, PMID: 30049474

[5] Loupy A Lefaucheur C . Antibody-mediated rejection of solid-organ allografts. N Engl J Med (2018) 379:1150−60. doi: 10.1056/NEJMra1802677, PMID: 30231232

[6] Pfirmann P Garrigue I Chauveau B Rondeau V Tumiotto C Weinmann L . Trends in epidemiology and risk factors of opportunistic infections in kidney transplant recipients between 2004-2022. Nephrol Dial Transplant. (2023) 39(4):627–636. doi: 10.1093/ndt/gfad193, PMID: 37667539

[7] Attias P Melica G Boutboul D De Castro N Audard V Stehlé T . Epidemiology, risk factors, and outcomes of opportunistic infections after kidney allograft transplantation in the era of modern immunosuppression: A monocentric cohort study. J Clin Med (2019) 8:594. doi: 10.3390/jcm8050594, PMID: 31052269 PMC6572426

[8] Echterdiek F Latus J Schwenger V . Immunosuppression in sensitized recipients. Curr Opin Organ Transplant. (2020) 25:80−5. doi: 10.1097/MOT.0000000000000721, PMID: 31815787

[9] Roberts MB Fishman JA . Immunosuppressive agents and infectious risk in transplantation: managing the « Net state of immunosuppression ». Clin Infect Dis (2021) 73:e1302−17. doi: 10.1093/cid/ciaa1189, PMID: 32803228 PMC8561260

[10] Aubert O Loupy A Hidalgo L Duong van Huyen JP Higgins S Viglietti D . Antibody-mediated rejection due to preexisting versus *de novo* donor-specific antibodies in kidney allograft recipients. J Am Soc Nephrol (2017) 28:1912−23. doi: 10.1681/ASN.2016070797, PMID: 28255002 PMC5461792

[11] Ling X Xiong J Liang W Schroder PM Wu L Ju W . Can immune cell function assay identify patients at risk of infection or rejection? A meta-analysis. Transplantation. (2012) 93:737. doi: 10.1097/TP.0b013e3182466248, PMID: 22357178

[12] Huskey J Gralla J Wiseman AC . Single time point immune function assay (ImmuKnow) testing does not aid in the prediction of future opportunistic infections or acute rejection. Clin J Am Soc Nephrol (2011) 6:423−9. doi: 10.2215/CJN.04210510, PMID: 21088287 PMC3052235

[13] Crepin T Gaiffe E Courivaud C Roubiou C Laheurte C Moulin B . Pre-transplant end-stage renal disease-related immune risk profile in kidney transplant recipients predicts post-transplant infections. Transpl Infect Dis (2016) 18:415−22. doi: 10.1111/tid.12534, PMID: 27027787

[14] DeWolfe D Aid M McGann K Ghofrani J Geiger E Helzer C . NK cells contribute to the immune risk profile in kidney transplant candidates. Front Immunol (2019) 10:1890/full. doi: 10.3389/fimmu.2019.01890/full, PMID: 31507586 PMC6716214

[15] Snanoudj R Candon S Roelen DL Jais JP Claas FH Legendre C . Peripheral B-cell phenotype and BAFF levels are associated with HLA immunization in patients awaiting kidney transplantation. Transplantation. (2014) 97:917−24. doi: 10.1097/01.TP.0000438211.34842.5e, PMID: 24827764

[16] Augusto JF Garnier AS Demiselle J Langs V Picquet J Legall R . Hypogammaglobulinemia and risk of severe infection in kidney transplant recipients. Transpl Infect Dis (2016) 18:741−51. doi: 10.1111/tid.12593, PMID: 27509578

[17] Loupy A Mengel M Haas M . Thirty years of the International Banff Classification for Allograft Pathology: the past, present, and future of kidney transplant diagnostics. Kidney Int (2022) 101:678−91. doi: 10.1016/j.kint.2021.11.028, PMID: 34922989

[18] Kahan RH Abraham N Lee HJ Ettenger RB Grimm PC Reed EF . Natural killer cell phenotypes and clinical outcomes in pediatric kidney transplantation. Pediatr Transplant. (2024) 28:e14877. doi: 10.1111/petr.14877, PMID: 39508125

[19] Campos C Pera A Lopez-Fernandez I Alonso C Tarazona R Solana R . Proinflammatory status influences NK cells subsets in the elderly. Immunol Lett nov (2014) 162:298−302. doi: 10.1016/j.imlet.2014.06.015, PMID: 24998470

[20] Gounder SS Abdullah BJJ Radzuanb NEIBM Zain FDBM Sait NBM Chua C . Effect of aging on NK cell population and their proliferation at ex vivo culture condition. Anal Cell Pathol (Amst). (2018) 2018:7871814. doi: 10.1155/2018/7871814, PMID: 30175033 PMC6098903

[21] Przemska-Kosicka A Childs CE Maidens C Dong H Todd S Gosney MA . Age-related changes in the natural killer cell response to seasonal influenza vaccination are not influenced by a synbiotic: a randomised controlled trial. Front Immunol (2018) 9:591. doi: 10.3389/fimmu.2018.00591, PMID: 29662493 PMC5890114

[22] Diebold M Vietzen H Heinzel A Haindl S Herz CT Mayer K . Natural killer cell functional genetics and donor-specific antibody-triggered microvascular inflammation. Am J Transplant. (2024) 24:743−54. doi: 10.1016/j.ajt.2023.12.005, PMID: 38097018

[23] Cook KD Waggoner SN Whitmire JK . NK cells and their ability to modulate T cells during virus infections. Crit Rev Immunol (2014) 34:359−88. doi: 10.1615/CritRevImmunol.2014010604, PMID: 25404045 PMC4266186

[24] Daniels KA O’Donnell CL Castonguay C Strutt TM McKinstry KK Swain SL . Virus-induced natural killer cell lysis of T cell subsets. Virology. (2020) 539:26−37. doi: 10.1016/j.virol.2019.10.003, PMID: 31670188 PMC7553765

[25] Lang PA Lang KS Xu HC Grusdat M Parish IA Recher M . Natural killer cell activation enhances immune pathology and promotes chronic infection by limiting CD8+ T-cell immunity. Proc Natl Acad Sci U.S.A (2012) 109:1210−5. doi: 10.1073/pnas.1118834109, PMID: 22167808 PMC3268324

[26] Cerboni C Zingoni A Cippitelli M Piccoli M Frati L Santoni A . Antigen-activated human T lymphocytes express cell-surface NKG2D ligands via an ATM/ATR-dependent mechanism and become susceptible to autologous NK- cell lysis. Blood. (2007) 110:606−15. doi: 10.1182/blood-2006-10-052720, PMID: 17405908

[27] Gjertsson I McGrath S Grimstad K Jonsson CA Camponeschi A Thorarinsdottir K . A close-up on the expanding landscape of CD21-/low B cells in humans. Clin Exp Immunol (2022) 210:217−29. doi: 10.1093/cei/uxac103, PMID: 36380692 PMC9985162

[28] Yam-Puc JC Hosseini Z Horner EC Gerber PP Beristain-Covarrubias N Hughes R . Age-associated B cells predict impaired humoral immunity after COVID-19 vaccination in patients receiving immune checkpoint blockade. Nat Commun 27 juin (2023) 14:3292. doi: 10.1038/s41467-023-38810-0, PMID: 37369658 PMC10299999

[29] Berger SP Sommerer C Witzke O Tedesco H Chadban S Mulgaonkar S . Two-year outcomes in de novo renal transplant recipients receiving everolimus-facilitated calcineurin inhibitor reduction regimen from the TRANSFORM study. Am J Transplant. (2019) 19:3018−34. doi: 10.1111/ajt.15480, PMID: 31152476

