# Supplementary material for: Skewed pretransplant lymphocytes subpopulations correlate with opportunistic infection onset within the first two years following kidney transplantation
Source: Front Immunol. 2025 Nov 6;16:1684313. doi: 10.3389/fimmu.2025.1684313 (PMC12631297; doi:10.3389/fimmu.2025.1684313)
Supplement: Supplementary Figure 1 — Blood flow gating strategy [file DataSheet1.docx]

**Supporting information**

1. **Supplemental figures**

**Supplemental Figure S.1: Blood flow cytometry gating strategy**


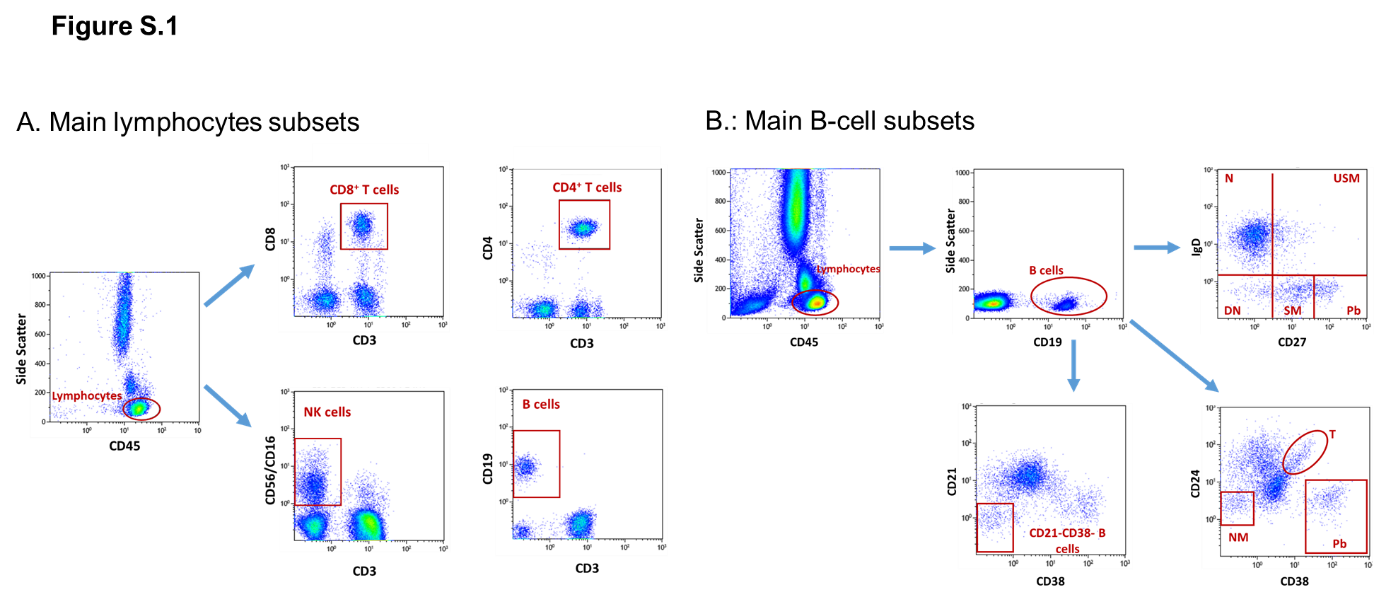


**Blood flow cytometry gating strategy. A.** Main lymphocytes subsets. Lymphocytes are gated on low-side scatter CD45+ cells. Using CD3+, CD4+ and CD8 markers allowed defining T CD3+, T CD4+ and T CD8+ lymphocytes. B cells are defined as CD3-CD19+ lymphocytes and NK cells are defined as CD3- CD56+/-CD16+/-. **B.** Main B cells subsets. B cells are gated on CD19+ lymphocytes. The different B-cell subsets were defined and gated on the basis of their expression of IgD and CD27, along with CD38, CD21 and CD24. N: naïve (IgD+ CD27-), USM: unswitched memory (IgD+ CD27+), SM: switched memory (IgD- CD27+), DN: double-negative (IgD- CD27-), T: transitional B cells (CD24++CD38++), NM: New Memory B cells (CD24-CD38-), Pb: plasmablast (CD24-CD38++ CD27++).

1. **Supplemental data**

OI definition

Infections caused by uncommon pathogens or by common pathogens with unusual and more severe forms, especially if modification of the immunosuppressive treatment is required for management of the infection.

The following pathogens, diseases or syndromes were included:

- Bacteria: Listeria monocytogenes (central nervous system [CNS] involvement); Nocardia sp. (CNS, lung, extensive cutaneous and/or disseminated disease ≥2 sites); Legionella pneumophila pneumonia; Mycobacterium tuberculosis (CNS, extra-pulmonary and extensive pulmonary involvement) and non-tuberculous mycobacteria (diagnosis criteria according to international guidelines).

- Virus:

severe herpes simplex virus (HSV) infections including encephalitis, pneumonitis or other organ involvement;

varicella-zoster virus (VZV) infections including encephalitis, pneumonitis, extensive shingles and zoster requiring appropriate antiviral treatment and alleviation of immunosuppression);

hepatitis B (HBV) reactivation, hepatitis E (HEV);

CMV syndrome (defined by the association of fever, malaise and leukopenia and/or thrombocytopenia) and end-organ disease (including pneumonia (CMV-DNA in bronchoalveolar lavage), central nervous system disease (CMV in cerebrospinal fluid), colitis, retinitis (confirmed by an ophthalmologist) or cholangitis);

Human-Herpes virus 8 (HHV8) associated Kaposi sarcoma;

JC virus-associated progressive multifocal leukoencephalopathy (PML);

BK virus-associated nephropathy (BKVN) as proven by allograft biopsy.

Norovirus (acute infection defined by symptoms including nausea, vomiting, diarrhea and positive stool sample by polymerase chain reaction analysis; chronic infection defined as the persistence of symptoms for ≥4 weeks or the necessity of rehospitalization because of norovirus-ascribed diarrhea, in combination with positive PCR results in at least one subsequent stool sample) and disseminated Adenovirus disease;

- Fungi:

invasive candidiasis, severe oropharyngeal candidiasis and esophagitis;

rare yeast such as Trichosporon spp.;

Cryptococcus neoformans (including meningitis, fungemia, disseminated infection involving ≥2 sites);

proven or probable invasive mold diseases (Aspergillus sp, Mucorales, Fusarium sp.);

invasive infections caused by dimorphic fungi such as Histoplasma capsulatum;

Pneumocystis jirovecii pneumonia.

- Parasites: Toxoplasma gondii (central nervous system involvement); Microsporidium sp, Cryptosporidium sp associated with chronic diarrhea (>1 month); visceral leishmania.

1. **Supplemental tables**

Supplemental Table S. 1: List of clones and providers of antibodies used for flow cytometry analysis

| **Antigen** | **clone** | **Fluorochrome** | **Manufacturer** |
| --- | --- | --- | --- |
| CD45 | B3821F4A | FITC | Beckman Coulter |
| CD45 | J33 | Krome Orange | Beckman Coulter |
| CD3 | UCHT1 | PC5 | Beckman Coulter |
| CD4 | SFCI12T4D11 | RD1 | Beckman Coulter |
| CD8 | SFCI21Thy2D3 | ECD | Beckman Coulter |
| CD19 | J3-119 | PC7 | Beckman Coulter |
| CD19 | J3-119 | ECD | Beckman Coulter |
| CD16 | 3G8 | RD1 | Beckman Coulter |
| CD56 | N901 | RD1 | Beckman Coulter |
| CD27 | 1A4CD27 | PC7 | Beckman Coulter |
| CD24 | ALB9 | APC | Beckman Coulter |
| CD38 | LS198-4-3 | APC-Alexa Fluor 750 | Beckman Coulter |
| CD21 | BL13 | PE | Beckman Coulter |
| IgD | IA6-2 | FITC | Beckman Coulter |
| IgM | SA-DA4 | PB | Beckman Coulter |

Supplemental Table S. 2 : Comparison of the two groups with and without available biological sample for immune analysis

| **Variables** | Whole cohort | No sample | Sample available | p-value |
| --- | --- | --- | --- | --- |
| **Patients, N (%)** | 422 | 139 | 283 |  |
| **Recipients characteristics** |  |  |  |  |
| Age, years, mean, SD | 54.5 (14.5) | 54.6 (15.5) | 54.4 (14.1) | 0.904 |
| Sex, Female, N (%) | 157 (37.2) | 54 (38.8) | 103 (36.4) | 0.702 |
| Diabetes before KT, N (%) | 111 (26.3) | 39 (28.1) | 72 (25.4) | 0.648 |
| Dialysis, N (%) | 380 (91.1) | 126 (92.0) | 254 (90.7) | 0.810 |
| Hemodialysis, N (%) | 348 (91.6) | 116 (92.1) | 232 (91.3) | 0.965 |
| **Donor characteristics** |  |  |  |  |
| Living donor, N (%) | 53 (12.6) | 15 (10.8) | 38 (13.4) | 0.541 |
| Extended Criteria Donor, N (%) | 215 (50.9) | 73 (52.5) | 142 (50.2) | 0.727 |
| Age, years, mean, SD | 57.4 (15.9) | 58.1 (17.0) | 57.0 (15.3) | 0.516 |
| eGFR, ml/min/1.73m², median [IQR] | 89.5 [65.9;105.3] | 86.1 [65.2;101.7] | 91.6 [67.2;105.9] | 0.273 |
| Immunosuppressive therapy |  |  |  |  |
| Induction, N (%) | 419 (99.3) | 137 (98.6) | 282 (99.6) | 0.253 |
| Basiliximab, N (%) | 154 (36.5) | 46 (33.1) | 108 (38.2) | 0.363 |
| Antithymocyte globulin, N (%) | 265 (62.8) | 91 (65.5) | 174 (61.5) | 0.491 |
| Maintenance |  |  |  |  |
| Calcineurin inhibitors, N (%) | 414 (98.1) | 134 (96.4) | 280 (98.9) | 0.122 |
| Mycophenolate mofetil, N (%) | 297 (70.4) | 102 (73.4) | 195 (68.9) | 0.405 |
| Belatacept, N (%) | 10 (2.4) | 5 (3.6) | 5 (1.8) | 0.309 |
| **Initial nephropathy** |  |  |  |  |
| Chronic interstitial nephropathy, N (%) | 17 (4.0) | 8 (5.8) | 9 (3.2) | 0.932 |
| Diabetes Mellitus, N (%) | 71 (16.8) | 24 (17.3) | 47 (16.6) |  |
| Genetic, N (%) | 72 (17.1) | 26 (18.7) | 46 (16.3) |  |
| Glomerulopathy, N(%) | 108 (25.6) | 33 (23.7) | 75 (26.5) |  |
| Hypertension, N (%) | 26 (6.2) | 8 (5.8) | 18 (6.4) |  |
| Other, N (%) | 20 (4.7) | 6 (4.3) | 14 (4.9) |  |
| Unknown, N (%) | 90 (21.3) | 28 (20.1) | 62 (21.9) |  |
| Urologic, N (%) | 18 (4.3) | 6 (4.3) | 12 (4.2) |  |
| **Kidney transplant characteristics** |  |  |  |  |
| Cold ischemia time, hours, median [IQR] | 15.9 [12.1;21.0] | 16.8 [13.0;21.3] | 15.4 [11.6;20.5] | 0.107 |
| Delayed graft function, N (%) | 123 (29.1) | 41 (29.5) | 82 (29) | 1.000 |


Supplemental Table S.3 : Interactions between variables and DSA at the time of KT

| **Variable*** | p-value control vs.OI | p-value control vs. AR |  |
| --- | --- | --- | --- |
|  |  |  |  |
| **Clinical characteristics** |  |  |  |
| *Recipient* |  |  |  |
| Age, years | 0.5551 | 0.7093 |  |
| Sex, Female | 0.6572 | 0.2481 |  |
| Diabetes before transplantation | 0.6684 | 0.3012 |  |
| Dialysis | 0.4439 | 0.2496 |  |
| Hemodialysis | 0.4482 | 0.924 |  |
| HIV+ | 0.074 | 0.2743 |  |
| HCV+ | 0.3077 | 0.8188 |  |
| *Donor* |  |  |  |
| Age, years | 0.3688 | 0.5649 |  |
| Living | 0.8677 | 0.2812 |  |
| Extended Criteria Donor | 0.283 | 0.952 |  |
| eGFR, ml/min/1.73m² | 0.6268 | 0.4883 |  |
| *Kidney transplantation* |  |  |  |
| CMV donor/recipient status | 0.6788 | 0.969 |  |
| Cold ischemia time, hours | 0.5561 | 0.7284 |  |
| Delayed graft function | 0.3053 | 0.1924 |  |
| Immunosuppressive therapy |  |  |  |
| Induction |  |  |  |
| Basiliximab | 0.9867 | 0.2216 |  |
| Antithymocyte globulin | 0.9686 | 0.2271 |  |
| Maintenance |  |  |  |
| Calcineurin inhibitors | NA | NA |  |
| Mycophenolate mofetil | 0.5839 | 0.28 |  |
| mTOR inhibitors | 0.6637 | 0.28 |  |
| Belatacept | 1 | 0.459 |  |
| **Biological characteristics** |  |  |  |
| **B cells** |  |  |  |
| Total, CD19+ |  |  |  |
| Absolute number, median [IQR] | 0.3268 | 0.4186 |  |
| Percentage, median [IQR] | 0.2797 | 0.4314 |  |
| Double negative memory (IgD- CD27-) |  |  |  |
| Absolute number, median [IQR] | 0.8702 | 0.7919 |  |
| Percentage, median [IQR] | 0.3385 | 0.2684 |  |
| Unswitched memory (IgD+ CD27+) |  |  |  |
| Absolute number, median [IQR] | 0.9797 | 0.9822 |  |
| Percentage, median [IQR] | 0.1154 | 0.1564 |  |
| Switched memory (IgD- CD27+) |  |  |  |
| Absolute number, median [IQR] | 0.8928 | 0.6828 |  |
| Percentage, median [IQR] | 0.4598 | 0.2615 |  |
| Naïve (IgD+ CD27-) |  |  |  |
| Absolute number, median [IQR] | 0.2677 | 0.1727 |  |
| Percentage, median [IQR] | 0.3333 | 0.1926 |  |
| Transitional (CD24high CD38high) |  |  |  |
| Absolute number, median [IQR] | 0.5949 | 0.2382 |  |
| Percentage, median [IQR] | 0.7524 | 0.3219 |  |
| Plasmablasts (CD24- CD38high CD27high) |  |  |  |
| Absolute number, median [IQR] | 0.3567 | 0.1858 |  |
| Percentage, median [IQR] | 0.8323 | 0.5442 |  |
| New memory |  |  |  |
| Absolute number, median [IQR] | 0.35 | 0.9521 |  |
| Percentage, median [IQR] | 0.4593 | 0.8381 |  |
| CD21- CD38- |  |  |  |
| Absolute number, median [IQR] | 0.5953 | 0.7254 |  |
| Percentage, median [IQR] | 0.0674 | 0.4451 |  |
| **T cells** |  |  |  |
| CD3+ |  |  |  |
| Absolute number, median [IQR] | 0.7228 | 0.5411 |  |
| Percentage, median [IQR] | 0.2899 | 0.6438 |  |
| CD4+ |  |  |  |
| Absolute number, median [IQR] | 0.1366 | 0.4409 |  |
| Percentage, median [IQR] | 0.1815 | 0.8667 |  |
| CD8+ |  |  |  |
| Absolute number, median [IQR] | 0.639 | 0.8425 |  |
| Percentage, median [IQR] | 0.1293 | 0.5584 |  |
| CD4/CD8 | 0.0815 | 0.7042 |  |
| NK cells |  |  |  |
| Absolute number, median [IQR] | 0.8681 | 0.6488 |  |
| Percentage, median [IQR] | 0.6617 | 0.9944 |  |

*Almost all biological variables have undergone mathematical transformations due to a lack of respect for normality (Log or SquareRoot transformations)

**Values in bold represent those with interaction values below 5%, but with the Bonferoni correction (threshold becomes 0.025) no interaction is significant.

Supplemental Table S. 4 : clinical and biological (immunologic cells) characteristics at the time of KT of recipients with AR or OI occurring before three months after KT

| **Variables** | Control | First acute rejection before three months after kidney transplantation | First Opportunistic infection before three months after kidney transplantation | p-value control vs. AR | p-value control vs.OI |
| --- | --- | --- | --- | --- | --- |
| **Patients, N (%)** | 190 | 23 | 17 |  |  |
| **Clinical data** | | | | | |
| **Recipient characteristics** |  |  |  |  |  |
| Age, years | 53.3 [43.0;64.6] | 56.6 [38.5;66.7] | 55.3 [53.4;63.6] | 0.940 | 0.525 |
| Sex, Female | 64 (33.7) | 11 (47.8) | 6 (35.3) | 0.801 | 1.000 |
| Diabetes before T | 46 (24.2) | 3 (13.0) | 4 (23.5) | 0.647 | 1.000 |
| Dialysis | 165 (88.2) | 22 (95.7) | 16 (94.1) | 1.000 | 1.000 |
| Hemodialysis | 149 (90.3) | 20 (90.9) | 16 (100) | 1.000 | 0.749 |
| **Donor characteristics** |  |  |  |  |  |
| Living donor | 30 (15.8) | 1 (4.35) | 2 (11.8) | 0.635 | 1.000 |
| Extended Criteria Donor | 89 (46.8) | 10 (43.5) | 10 (58.8) | 0.933 | 0.784 |
| Donor age | 56.5 [46.0;65.0] | 56.0 [52.0;65.5] | 62.0 [50.0;64.0] | 0.670 | 0.670 |
| eGFR, ml/min/1.73m² | 92.8 [67.7;106] | 86.4 [45.7;111] | 84.9 [71.7;103] | 0.859 | 0.859 |
| **Kidney transplant characteristics** |  |  |  |  |  |
| Donor specific anti-HLA antibodies | 41 (22.3) | 6 (27.3) | 6 (35.3) | 0.851 | 0.711 |
| Delayed graft function | 48 (25.3) | 9 (39.1) | 7 (41.2) | 0.363 | 0.363 |
| Immunosuppressive therapy |  |  |  |  |  |
| Induction | 189 (99.5) | 23 (100) | 17 (100) | 1.000 | 1.000 |
| Basiliximab | 77 (40.5) | 9 (39.1) | 8 (47.1) | 1.000 | 1.000 |
| Antithymocyte globulin | 112 (58.9) | 14 (60.9) | 9 (52.9) | 1.000 | 1.000 |
| Maintenance |  |  |  |  |  |
| Calcineurin inhibitors | 188 (98.9) | 22 (95.7) | 17 (100) | 0.874 | 1.000 |
| Mycophenolate mofetil | 125 (65.8) | 17 (73.9) | 14 (82.4) | 0.707 | 0.707 |
| Belatacept | 4 (2.11) | 1 (4.35) | 0 (0.00) | 1.000 | 1.000 |
| mtor | 65 (34.2) | 6 (26.1) | 4 (23.5) | 0.877 | 0.877 |
| Steroids | 190 (100) | 23 (100) | 17 (100) |  |  |
| **Within 12 months after transplantation** |  |  |  |  |  |
| Kidney allograft loss, N (%) | 2 (1.05) | 0 (0.00) | 0 (0.00) | 1.000 | 1.000 |
| Patient death, N (%) | 10 (5.26) | 2 (8.70) | 1 (5.88) | 1.000 | 1.000 |
| eGFR, ml/min/1.73m², median [IQR] | 50.0 [35.9;60.4] | 38.1 [23.5;49.2] | 38.2 [29.1;48.1] | 0.012 | 0.034 |
| **Within 24 months after transplantation** |  |  |  |  |  |
| Kidney allograft loss, N (%) | 4 (2.11) | 1 (4.35) | 0 (0.00) | 1.000 | 1.000 |
| Patient death, N (%) | 18 (9.47) | 3 (13.0) | 1 (5.88) | 0.935 | 1.000 |
| eGFR, ml/min/1.73m², median [IQR] | 48.7 [37.2;60.0] | 42.8 [24.1;49.9] | 36.3 [30.0;52.3] | 0.080 | 0.147 |
| **Biological data** | | | | | |
| **B cells** |  |  |  |  |  |
| Total |  |  |  |  |  |
| Absolute number, median [IQR] | 106 [70.0;176] | 144 [76.5;204] | 107 [92.0;200] | 0.684 | 0.684 |
| Percentage, median [IQR] | 9.65 [6.38;13.7] | 11.2 [7.90;13.4] | 10.2 [6.15;17.8] | 0.875 | 0.875 |
| Double negative memory (IgD- CD27-) |  |  |  |  |  |
| Absolute number, median [IQR] | 3.92 [2.24;7.12] | 4.21 [3.09;5.44] | 7.51 [2.35;12.1] | 0.657 | 0.473 |
| Percentage, median [IQR] | 3.76 [2.20;5.84] | 3.64 [2.53;4.82] | 3.30 [2.34;5.32] | 0.925 | 0.925 |
| Unswitched memory (IgD+ CD27+) |  |  |  |  |  |
| Absolute number, median [IQR] | 14.3 [8.57;22.4] | 16.9 [10.9;24.3] | 14.3 [10.1;25.8] | 0.917 | 0.917 |
| Percentage, median [IQR] | 13.0 [8.56;17.8] | 11.2 [8.73;24.2] | 11.9 [9.97;15.2] | 0.985 | 0.985 |
| Switched memory (IgD- CD27+) |  |  |  |  |  |
| Absolute number, median [IQR] | 15.5 [9.54;24.8] | 14.5 [9.77;29.0] | 23.6 [9.81;38.3] | 0.890 | 0.476 |
| Percentage, median [IQR] | 15.3 [8.85;24.5] | 10.5 [8.32;24.4] | 14.6 [8.95;21.1] | 0.842 | 0.842 |
| Naïve (IgD+ CD27-) |  |  |  |  |  |
| Absolute number, median [IQR] | 67.2 [36.2;123] | 70.1 [50.4;145] | 79.1 [46.6;157] | 0.540 | 0.540 |
| Percentage, median [IQR] | 65.5 [51.2;77.3] | 71.4 [47.9;78.1] | 69.6 [59.7;79.9] | 0.869 | 0.869 |
| Transitional (CD24high CD38high) |  |  |  |  |  |
| Absolute number, median [IQR] | 4.60 [2.30;10.8] | 5.38 [2.66;13.9] | 5.96 [2.79;16.4] | 0.575 | 0.575 |
| Percentage, median [IQR] | 4.59 [2.68;7.08] | 6.32 [2.59;9.10] | 6.05 [3.41;9.41] | 0.656 | 0.656 |
| Plasmablasts (CD24- CD38high CD27high) |  |  |  |  |  |
| Absolute number, median [IQR] | 1.39 [0.70;2.50] | 1.20 [0.71;2.92] | 2.09 [1.08;4.16] | 0.914 | 0.221 |
| Percentage, median [IQR] | 1.29 [0.66;2.51] | 1.16 [0.66;1.78] | 1.40 [1.00;2.35] | 0.648 | 0.648 |
| New memory (CD24- CD38low) |  |  |  |  |  |
| Absolute number, median [IQR] | 1.85 [0.93;3.41] | 2.12 [1.03;4.06] | 3.70 [0.91;6.39] | 0.719 | 0.599 |
| Percentage, median [IQR] | 1.65 [0.92;3.39] | 1.75 [1.00;2.82] | 1.73 [1.08;2.86] | 0.986 | 0.986 |
| CD21- CD38- |  |  |  |  |  |
| Absolute number, median [IQR] | 2.28 [1.12;4.21] | 2.77 [1.91;4.94] | 3.12 [1.66;5.56] | 0.440 | 0.440 |
| Percentage, median [IQR] | 1.86 [1.10;4.30] | 2.36 [1.02;4.00] | 2.26 [1.07;3.02] | 0.790 | 0.790 |
| **T cells** |  |  |  |  |  |
| CD3+ |  |  |  |  |  |
| Absolute number, median [IQR] | 846 [646;1124] | 830 [640;1293] | 935 [728;1096] | 0.977 | 0.977 |
| Percentage, median [IQR] | 72.7 [64.6;80.0] | 70.9 [66.5;79.2] | 67.6 [62.9;73.0] | 0.939 | 0.243 |
| CD4+ |  |  |  |  |  |
| Absolute number, median [IQR] | 497 [381;711] | 582 [454;765] | 472 [422;602] | 0.602 | 0.764 |
| Percentage, median [IQR] | 43.9 [37.5;49.7] | 45.5 [41.0;49.0] | 39.3 [31.9;44.2] | 0.543 | 0.068 |
| CD8+ |  |  |  |  |  |
| Absolute number, median [IQR] | 292 [198;431] | 287 [209;446] | 338 [254;443] | 0.728 | 0.728 |
| Percentage, median [IQR] | 24.4 [19.8;32.1] | 25.7 [22.0;30.5] | 26.8 [22.8;30.5] | 0.943 | 0.943 |
| CD4/CD8 | 1.65 [1.26;2.46] | 1.57 [1.34;2.54] | 1.61 [1.27;1.95] | 0.860 | 0.697 |
| NK cells |  |  |  |  |  |
| Absolute number, median [IQR] | 187 [132;252] | 200 [130;272] | 282 [257;396] | 0.791 | 0.002 |
| Percentage, median [IQR] | 15.3 [9.62;22.3] | 16.9 [8.24;23.1] | 19.6 [18.0;23.3] | 0.965 | 0.102 |

Supplemental table S.5: clinical and biological (immune cells) characteristics at the time of KT of recipients with acute rejection or opportunistic infection occurring after three months

| **Variables** | Control | First acute rejection after three months after kidney transplantation | First Opportunistic infection after three months after kidney transplantation | p-value control vs. AR | p-value control vs.OI |
| --- | --- | --- | --- | --- | --- |
| **Patients, N (%)** | 190 | 21 | 32 |  |  |
| **Clinical data** | | | | | |
| **Recipient characteristics** |  |  |  |  |  |
| Age, years | 53.3 [43.0;64.6] | 55.8 [46.0;67.5] | 60.0 [49.3;69.0] | 0.388 | 0.095 |
| Sex, Female | 64 (33.7) | 6 (28.6) | 16 (50.0) | 0.820 | 0.310 |
| Diabetes before T | 46 (24.2) | 8 (38.1) | 11 (34.4) | 0.477 | 0.477 |
| Dialysis | 165 (88.2) | 19 (90.5) | 32 (100) | 1.000 | 0.152 |
| Hemodialysis | 149 (90.3) | 16 (84.2) | 31 (96.9) | 0.422 | 0.422 |
| **Donor characteristics** |  |  |  |  |  |
| Living donor | 30 (15.8) | 2 (9.52) | 3 (9.38) | 1.000 | 1.000 |
| Extended Criteria Donor | 89 (46.8) | 13 (61.9) | 20 (62.5) | 0.420 | 0.420 |
| Donor age | 56.5 [46.0;65.0] | 62.0 [50.0;74.0] | 62.0 [52.8;71.0] | 0.176 | 0.120 |
| eGFR, ml/min/1.73m² | 92.8 [67.7;106] | 90.9 [75.6;107] | 92.0 [70.9;104] | 0.810 | 0.810 |
| **Kidney transplant characteristics** |  |  |  |  |  |
| Donor specific anti-HLA antibodies | 41 (22.3) | 4 (20.0) | 8 (26.7) | 1.000 | 1.000 |
| Delayed graft function | 48 (25.3) | 6 (28.6) | 12 (37.5) | 0.947 | 0.660 |
| Immunosuppressive therapy |  |  |  |  |  |
| Induction | 189 (99.5) | 21 (100) | 32 (100) | 1.000 | 1.000 |
| Basiliximab | 77 (40.5) | 7 (33.3) | 7 (21.9) | 0.686 | 0.208 |
| Antithymocyte globulin | 112 (58.9) | 14 (66.7) | 25 (78.1) | 0.653 | 0.185 |
| Maintenance |  |  |  |  |  |
| Calcineurin inhibitors | 188 (98.9) | 21 (100) | 32 (100) | 1.000 | 1.000 |
| Mycophenolate mofetil | 125 (65.8) | 14 (66.7) | 25 (78.1) | 1.000 | 0.720 |
| Belatacept | 4 (2.11) | 0 (0.00) | 0 (0.00) | 1.000 | 1.000 |
| mtor | 65 (34.2) | 7 (33.3) | 7 (21.9) | 1.000 | 0.720 |
| Steroids | 190 (100) | 21 (100) | 32 (100) | . | . |
| **Within 12 months after transplantation** |  |  |  |  |  |
| Kidney allograft loss, N () | 2 (1.05) | 1 (4.76) | 1 (3.12) | 0.562 | 0.562 |
| Patient death, N () | 10 (5.26) | 0 (0.00) | 0 (0.00) | 0.603 | 0.603 |
| eGFR, ml/min/1.73m², median [IQR] | 50.0 [35.9;60.4] | 37.9 [27.9;49.6] | 33.4 [28.0;47.7] | 0.044 | 0.004 |
| **Within 24 months after transplantation** |  |  |  |  |  |
| Kidney allograft loss, N () | 4 (2.11) | 2 (9.52) | 2 (6.25) | 0.313 | 0.313 |
| Patient death, N () | 18 (9.47) | 0 (0.00) | 4 (12.5) | 0.340 | 0.533 |
| eGFR, ml/min/1.73m², median [IQR] | 48.7 [37.2;60.0] | 38.6 [21.4;56.0] | 37.8 [20.0;55.1] | 0.060 | 0.060 |
| **Biological data** | | | | | |
| **B cells** |  |  |  |  |  |
| Total |  |  |  |  |  |
| Absolute number, median [IQR] | 106 [70.0;176] | 105 [74.0;190] | 130 [72.0;222] | 0.790 | 0.790 |
| Percentage, median [IQR] | 9.65 [6.38;13.7] | 9.10 [7.10;14.3] | 11.8 [7.72;16.1] | 0.759 | 0.659 |
| Double negative memory (IgD- CD27-) |  |  |  |  |  |
| Absolute number, median [IQR] | 3.92 [2.24;7.12] | 2.75 [1.51;5.95] | 3.73 [2.57;6.66] | 0.296 | 0.999 |
| Percentage, median [IQR] | 3.76 [2.20;5.84] | 3.03 [1.91;4.68] | 3.30 [2.14;5.69] | 0.571 | 0.704 |
| Unswitched memory (IgD+ CD27+) |  |  |  |  |  |
| Absolute number, median [IQR] | 14.3 [8.57;22.4] | 14.3 [7.45;21.8] | 11.0 [8.10;18.8] | 0.848 | 0.848 |
| Percentage, median [IQR] | 13.0 [8.56;17.8] | 11.1 [8.20;18.1] | 10.5 [7.92;13.7] | 0.975 | 0.334 |
| Switched memory (IgD- CD27+) |  |  |  |  |  |
| Absolute number, median [IQR] | 15.5 [9.54;24.8] | 14.2 [8.60;31.8] | 17.6 [12.0;24.8] | 0.876 | 0.876 |
| Percentage, median [IQR] | 15.3 [8.85;24.5] | 12.9 [10.2;22.5] | 15.2 [8.71;18.4] | 0.907 | 0.749 |
| Naïve (IgD+ CD27-) |  |  |  |  |  |
| Absolute number, median [IQR] | 67.2 [36.2;123] | 68.3 [51.3;106] | 77.5 [44.0;185] | 0.775 | 0.718 |
| Percentage, median [IQR] | 65.5 [51.2;77.3] | 72.6 [54.0;76.4] | 71.1 [62.6;79.1] | 0.757 | 0.656 |
| Transitional (CD24high CD38high) |  |  |  |  |  |
| Absolute number, median [IQR] | 4.60 [2.30;10.8] | 2.84 [1.23;8.61] | 6.60 [3.89;14.4] | 0.176 | 0.176 |
| Percentage, median [IQR] | 4.59 [2.68;7.08] | 3.30 [1.45;5.27] | 6.02 [3.83;7.44] | 0.055 | 0.245 |
| Plasmablasts (CD24- CD38high CD27high) |  |  |  |  |  |
| Absolute number, median [IQR] | 1.39 [0.70;2.50] | 1.80 [0.79;3.07] | 1.79 [1.11;2.94] | 0.828 | 0.318 |
| Percentage, median [IQR] | 1.29 [0.66;2.51] | 1.27 [0.78;2.20] | 1.77 [0.90;3.14] | 0.828 | 0.488 |
| New memory (CD24- CD38low) |  |  |  |  |  |
| Absolute number, median [IQR] | 1.85 [0.93;3.41] | 1.84 [1.05;2.50] | 2.00 [1.16;3.46] | 0.729 | 0.729 |
| Percentage, median [IQR] | 1.65 [0.92;3.39] | 1.68 [1.29;2.11] | 2.02 [0.96;3.37] | 0.923 | 0.923 |
| CD21- CD38- |  |  |  |  |  |
| Absolute number, median [IQR] | 2.28 [1.12;4.21] | 2.54 [1.35;3.62] | 2.96 [2.22;4.09] | 0.977 | 0.108 |
| Percentage, median [IQR] | 1.86 [1.10;4.30] | 1.88 [1.72;3.32] | 3.02 [1.46;4.31] | 0.858 | 0.525 |
| **T cells** |  |  |  |  |  |
| CD3+ |  |  |  |  |  |
| Absolute number, median [IQR] | 846 [646;1124] | 947 [689;1088] | 698 [628;1062] | 0.738 | 0.407 |
| Percentage, median [IQR] | 72.7 [64.6;80.0] | 75.0 [70.0;80.3] | 65.7 [58.0;73.8] | 0.374 | 0.006 |
| CD4+ |  |  |  |  |  |
| Absolute number, median [IQR] | 497 [381;711] | 575 [395;748] | 446 [356;612] | 0.430 | 0.241 |
| Percentage, median [IQR] | 43.9 [37.5;49.7] | 48.6 [41.4;54.5] | 38.5 [34.4;45.2] | 0.093 | 0.062 |
| CD8+ |  |  |  |  |  |
| Absolute number, median [IQR] | 292 [198;431] | 330 [170;467] | 282 [177;428] | 0.983 | 0.954 |
| Percentage, median [IQR] | 24.4 [19.8;32.1] | 25.5 [16.6;31.2] | 25.4 [16.9;32.8] | 0.785 | 0.785 |
| CD4/CD8 | 1.65 [1.26;2.46] | 2.04 [1.59;3.06] | 1.62 [1.15;2.27] | 0.345 | 0.616 |
| NK cells |  |  |  |  |  |
| Absolute number, median [IQR] | 187 [132;252] | 178 [110;191] | 204 [170;313] | 0.273 | 0.066 |
| Percentage, median [IQR] | 15.3 [9.62;22.3] | 14.3 [8.00;19.1] | 20.0 [14.0;24.9] | 0.426 | 0.032 |

Supplemental table S.6: Immune cells characteristics at M3 of recipients with acute rejection or opportunistic infection occurring after three months

| **Variables** | Control | First acute rejection after three months after kidney transplantation | First Opportunistic infection after three months after kidney transplantation | p-value three groups | p-value control vs. AR | p-value control vs.OI |
| --- | --- | --- | --- | --- | --- | --- |
| **Patients, N ()** | 159 | 18 | 27 |  |  |  |
| **B cells** |  |  |  |  |  |  |
| Total, CD19+ |  |  |  |  |  |  |
| Absolute number, median [IQR] | 103 [52.0;175] | 100 [72.2;163] | 72.0 [50.0;109] | 0.234 | 0.549 | 0.206 |
| Percentage, median [IQR] | 13.4 [8.15;21.8] | 15.2 [10.6;18.2] | 18.4 [8.30;25.1] | 0.659 | 0.948 | 0.596 |
| Double negative memory (IgD- CD27-) |  |  |  |  |  |  |
| Absolute number, median [IQR] | 3.28 [1.82;7.02] | 3.44 [1.80;4.96] | 2.62 [1.84;3.59] | 0.643 | 0.726 | 0.726 |
| Percentage, median [IQR] | 3.55 [2.39;5.23] | 2.89 [2.01;3.42] | 3.84 [2.14;5.20] | 0.230 | 0.230 | 0.874 |
| Unswitched memory (IgD+ CD27+) |  |  |  |  |  |  |
| Absolute number, median [IQR] | 11.1 [6.35;23.2] | 16.4 [7.67;35.5] | 8.99 [5.10;15.1] | 0.148 | 0.276 | 0.194 |
| Percentage, median [IQR] | 12.3 [8.23;17.1] | 14.4 [10.5;19.2] | 11.0 [7.56;14.3] | 0.333 | 0.337 | 0.337 |
| Switched memory (IgD- CD27+) |  |  |  |  |  |  |
| Absolute number, median [IQR] | 13.4 [6.41;24.1] | 16.4 [5.84;32.6] | 11.6 [7.19;18.6] | 0.733 | 0.674 | 0.674 |
| Percentage, median [IQR] | 14.4 [7.26;23.0] | 15.0 [8.71;19.0] | 18.6 [10.1;21.4] | 0.896 | 0.896 | 0.896 |
| Naïve (IgD+ CD27-) |  |  |  |  |  |  |
| Absolute number, median [IQR] | 60.6 [30.7;117] | 67.0 [47.9;113] | 57.4 [41.7;93.6] | 0.619 | 0.622 | 0.704 |
| Percentage, median [IQR] | 67.8 [53.5;80.1] | 64.4 [56.9;77.2] | 68.4 [60.6;78.5] | 0.910 | 0.780 | 0.780 |
| Transitional (CD24high CD38high) |  |  |  |  |  |  |
| Absolute number, median [IQR] | 0.23 [0.08;0.76] | 0.16 [0.10;0.59] | 0.18 [0.04;0.68] | 0.681 | 0.797 | 0.797 |
| Percentage, median [IQR] | 0.22 [0.08;0.67] | 0.15 [0.10;0.36] | 0.22 [0.08;0.48] | 0.747 | 0.741 | 0.741 |
| Plasmablasts (CD24- CD38high CD27high) |  |  |  |  |  |  |
| Absolute number, median [IQR] | 0.13 [0.06;0.36] | 0.08 [0.05;0.42] | 0.10 [0.06;0.31] | 0.801 | 0.932 | 0.932 |
| Percentage, median [IQR] | 0.14 [0.06;0.27] | 0.08 [0.04;0.28] | 0.12 [0.05;0.32] | 0.700 | 0.698 | 0.979 |
| New memory |  |  |  |  |  |  |
| Absolute number, median [IQR] | 1.15 [0.63;2.45] | 1.28 [0.81;2.55] | 1.13 [0.72;2.26] | 0.825 | 0.843 | 0.944 |
| Percentage, median [IQR] | 1.27 [0.62;2.61] | 1.13 [0.86;1.78] | 1.45 [0.78;2.95] | 0.629 | 0.917 | 0.726 |
| CD21- CD38- |  |  |  |  |  |  |
| Absolute number, median [IQR] | 1.80 [0.84;3.35] | 2.50 [1.20;3.91] | 1.72 [1.48;3.31] | 0.421 | 0.575 | 0.575 |
| Percentage, median [IQR] | 1.87 [0.91;4.06] | 1.98 [1.18;2.92] | 2.54 [1.77;4.22] | 0.260 | 0.749 | 0.320 |
| **T cells** |  |  |  |  |  |  |
| CD3+ |  |  |  |  |  |  |
| Absolute number, median [IQR] | 498 [267;864] | 592 [466;922] | 347 [148;538] | 0.010 | 0.456 | 0.013 |
| Percentage, median [IQR] | 69.2 [57.7;78.4] | 71.2 [64.3;78.8] | 61.7 [49.7;70.7] | 0.034 | 0.662 | 0.034 |
| CD4+ |  |  |  |  |  |  |
| Absolute number, median [IQR] | 250 [116;547] | 368 [114;625] | 155 [55.5;290] | 0.024 | 0.740 | 0.025 |
| Percentage, median [IQR] | 34.5 [24.7;46.2] | 33.9 [22.0;49.2] | 27.5 [18.8;39.9] | 0.148 | 0.940 | 0.151 |
| CD8+ |  |  |  |  |  |  |
| Absolute number, median [IQR] | 217 [130;318] | 330 [138;380] | 144 [55.0;230] | 0.005 | 0.365 | 0.005 |
| Percentage, median [IQR] | 27.8 [22.7;33.2] | 29.5 [21.7;34.8] | 29.5 [18.9;34.2] | 0.880 | 0.872 | 0.872 |
| CD4/CD8 | 1.25 [0.78;1.81] | 1.20 [0.90;1.81] | 1.17 [0.65;1.55] | 0.684 | 0.811 | 0.811 |
| NK cells |  |  |  |  |  |  |
| Absolute number, median [IQR] | 94.0 [65.0;146] | 108 [78.0;140] | 95.0 [41.0;149] | 0.694 | 0.609 | 0.609 |
| Percentage, median [IQR] | 12.4 [7.88;21.0] | 12.6 [8.80;18.6] | 15.9 [11.3;23.4] | 0.324 | 0.954 | 0.413 |
